# Prognostic relevance of Wnt-inhibitory factor-1 (*WIF1*) and Dickkopf-3 (*DKK3*) promoter methylation in human breast cancer

**DOI:** 10.1186/1471-2407-9-217

**Published:** 2009-07-01

**Authors:** Jürgen Veeck, Peter J Wild, Thomas Fuchs, Peter J Schüffler, Arndt Hartmann, Ruth Knüchel, Edgar Dahl

**Affiliations:** 1 Molecular Oncology Group, Institute of Pathology, University Hospital of the RWTH Aachen, Pauwelsstrasse 30, D-52074 Aachen, Germany; 2 Institute of Surgical Pathology, University Hospital Zürich, Schmelzbergstrasse 12, CH-8091, Zürich, Switzerland; 3 Institute for Computational Science and Department of Computer Science, ETH Zürich, Universitätsstrasse 6, CH-8092 Zürich, Switzerland; 4 Department of Pathology, University of Erlangen, Krankenhausstrasse 12, D-91054 Erlangen, Germany; 5 Current address: Cancer Epigenetics and Biology Program (PEBC), Bellvitge Institute for Biomedical Research (ICO-IDIBELL), Hospital Duran i Reynals, Av. Gran Via de L'Hospitalet 199-203, E-08907 Barcelona, Spain

## Abstract

**Background:**

Secreted Wnt signaling antagonists have recently been described as frequent targets of epigenetic inactivation in human tumor entities. Since gene silencing of certain Wnt antagonists was found to be correlated with adverse patient survival in cancer, we aimed at investigating a potential prognostic impact of the two Wnt antagonizing molecules *WIF1 *and *DKK3 *in breast cancer, which are frequently silenced by promoter methylation in this disease.

**Methods:**

*WIF1 *and *DKK3 *promoter methylation were assessed by methylation-specific PCR with bisulfite-converted DNA from 19 normal breast tissues and 150 primary breast carcinomas. Promoter methylation was interpreted in a qualitative, binary fashion. Statistical evaluations included two-sided Fisher's exact tests, univariate log-rank tests of Kaplan-Meier curves as well as multivariate Cox regression analyses.

**Results:**

*WIF1 and DKK3 *promoter methylation were detected in 63.3% (95/150) and 61.3% (92/150) of breast carcinoma samples, respectively. In normal breast tissues, *WIF1 *methylation was present in 0% (0/19) and *DKK3 *methylation in 5.3% (1/19) of samples. In breast carcinomas, *WIF1 *methylation was significantly associated with methylation of *DKK3 *(p = 0.009). Methylation of either gene was not associated with clinicopathological parameters, except for *DKK3 *methylation being associated with patient age (p = 0.007). In univariate analysis, *WIF1 *methylation was not associated with clinical patient outcome. In contrast, *DKK3 *methylation was a prognostic factor in patient overall survival (OS) and disease-free survival (DFS). Estimated OS rates after 10 years were 54% for patients with *DKK3*-methylated tumors, in contrast to patients without *DKK3 *methylation in the tumor, who had a favorable 97% OS after 10 years (p < 0.001). Likewise, DFS at 10 years for patients harboring *DKK3 *methylation in the tumor was 58%, compared with 78% for patients with unmethylated *DKK3 *(p = 0.037). Multivariate analyses revealed that *DKK3 *methylation was an independent prognostic factor predicting poor OS (hazard ratio (HR): 14.4; 95% confidence interval (CI): 1.9–111.6; p = 0.011), and short DFS (HR: 2.5; 95% CI: 1.0–6.0; p = 0.047) in breast cancer.

**Conclusion:**

Although the Wnt antagonist genes *WIF1 *and *DKK3 *show a very similar frequency of promoter methylation in human breast cancer, only *DKK3 *methylation proves as a novel prognostic marker potentially useful in the clinical management of this disease.

## Background

The most common epigenetic alteration in human cancer affecting gene expression is 5'-cytosine methylation within CpG islands in gene promoter regions [1]. Promoter methylation effectively represses RNA transcription and occurs in many genes involved in human cancer development [2]. The majority of these affected genes are potential or known tumor suppressor genes that are regulators of different cellular pathways, such as cell cycle, DNA repair, growth factor signaling or cell adhesion [3]. Wnt signaling is one of the central cellular pathways commonly disrupted in several tumor types, including breast cancer [4,5]. Unlike colorectal cancer, evidence for genetic alterations of Wnt pathway components in breast cancer, such as adenomatous polyposis coli (*APC*) mutations, is rare [6]. Several lines of evidence suggest that in breast cancer the Wnt signaling pathway is disrupted predominantly through epigenetic aberrations, most of all by promoter methylation of genes encoding secreted Wnt inhibitory molecules. For instance, genes encoding secreted frizzled-related proteins (SFRP) and Wnt-inhibitory factor-1 (*WIF1*) were previously reported as frequent targets of epigenetic inactivation in breast cancer [7-12]. In addition to this, we have recently shown that the putative Wnt signaling inhibitor Dickkopf-3 (*DKK3*) is functionally inactivated by promoter methylation in more than 60% of tumors from patients with invasive breast cancer [13]. Besides secreted inhibitors, two studies also reported frequent methylation of the *APC *gene in breast carcinomas [14,15]. Altogether, this provides strong evidence for an epigenetically disrupted and thereby activated Wnt signaling pathway in the development of human breast cancer.

There is increasing evidence that promoter methylation of cancer-related genes can be one of the most prevalent molecular markers for human cancer diseases [16]. The potential clinical applications of DNA-methylation biomarkers may include diagnosis of neoplasm, tumor classification, prediction of response to treatment, or patient prognosis [17]. Methylation of particular Wnt pathway genes has already been described as a potential biomarker for unfavorable patient outcome in human cancer. For instance, we have recently shown that methylation of *SFRP1 *as well as *SFRP5 *is associated with reduced patient overall survival in breast cancer [7,10]. In contrast to this, high-frequent methylation of *SFRP2 *was not prognostically relevant in breast cancer [9], but was shown to comprise a diagnostic value as a sensitive screening marker for the stool-based detection of colorectal cancer and premalignant colorectal lesions [18-20]. *DKK3 *methylation is associated with reduced DFS in acute lymphoblastic leukemia [21], and also with shorter OS in kidney cancer [22] and non-small cell lung cancer [23], as well as very recently reported with OS in gastric cancer [24]. Taken together, promoter methylation of Wnt signaling antagonists appears to provide a rich pool of novel tumor biomarkers in human cancer, potentially useful in the clinical setting by helping to improve management of this disease.

In the present study, we addressed the question to whether promoter methylation of two Wnt antagonist genes (*WIF1 *and *DKK3*), that were previously reported as hypermethylated in breast cancer, provides prognostically relevant information in this tumor entity. In univariate and multivariate analyses we have investigated gene methylation in a large cohort (n = 150) of invasive breast cancer specimens. We here demonstrate for the first time that *DKK3 *methylation, but not *WIF1 *methylation, is an independent prognostic factor indicating poor patient survival in human breast cancer.

## Methods

### Patient material

Surgically resected samples were obtained from 150 unselected breast cancer patients at the Departments of Gynecology at the University Hospitals of Aachen, Jena, Regensburg and Düsseldorf in Germany from 1991 to 2005. For 19 patients, normal breast tissues were available. In all cases, at least two-board certified pathologists agreed on the diagnosis on breast cancer. The samples were recruited in a non-selective, consecutive manner. Cases were not stratified for any known pre-operative or pathological prognostic factor. Inclusion criteria for the study were: Female patients presenting with unilateral, primary invasive breast cancer without individual breast cancer history. Exclusion criteria were: neo-adjuvant chemotherapy prior to surgery, presentation with secondary breast cancer, or peritumorous carcinoma *in situ *present in the tumor sample. All patients gave informed consent for retention and analysis of their tissue for research purposes and the Institutional Review Boards of the participating centers approved the study. Tumor histology was determined according to the criteria of the WHO (2003), while disease stage was assessed according to UICC [25]. Histological, tumors were graded according to Bloom and Richardson, as modified by Elston and Ellis [26]. Hormone receptor status was assessed according to the scoring system developed by Remmele and Stegner [27]. For 125 patients follow-up data were available with a median time of 64 months (range 1 to 174 months). Patient characteristics of this cohort have been previously described [13].

### Extraction of genomic DNA

Tumor material was snap-frozen in liquid nitrogen immediately after surgery. Hematoxylin/Eosin-stained sections were prepared for assessing the percentage of tumor cells; only samples with > 70% tumor cells were selected. A total of 20 tissue sections (20 μm each) per specimen was dissected in a cryotom and pooled. Normal breast tissue specimens were prepared likewise. For normal breast samples, the epithelial cell amount had to exceed 30% in order to be selected for further preparation. Samples were dissolved in lysis buffer followed by DNA isolation, using the QIAamp DNA Mini Kit (Qiagen, Hilden, Germany) according to the manufacturer's recommendations. The extracted genomic DNA was finally diluted in 55 μl of Tris buffer (10 mM; pH 7.6).

### *In silico *promoter analysis

The *WIF1 *promoter, located at chromosome position 12q14.2, was investigated according to the contig ENSG00000156076 contained in the Ensembl database [28]. A genomic nucleotide sequence consisting of 1000 bp upstream of the annotated transcription start site (TSS) and 293 bp downstream of the TSS (first exon) was analyzed by methprimer software [29]. Criteria for CpG island prediction were adjusted to the definition from Takai and Jones [30], and included an observed/expected CpG ratio of ≥ 0.7 and a GC content of ≥ 60%. The identified CpG island proximal to the TSS was chosen for promoter methylation analysis. Methylation-specific PCR (MSP) primers were derived from a particular region within this island, which has been analyzed for CpG methylation by bisulfite genomic sequencing (BGS) in a previous study [11]. The *DKK3 *promoter, located at chromosome position 11p15.3, was investigated according to the contig ENSG00000050165 contained in the Ensembl database. A genomic nucleotide sequence consisting of 1000 bp upstream of the annotated TSS and 1001 bp downstream of the TSS was analyzed by methprimer software. The downstream region covered the first three exons of the gene, since *DKK3 *was reported to be alternatively transcribed under the control of two distinct promoters [31]. Methprimer software identified the existence of two distinct CpG islands, located proximal to either of the two predicted transcription start sites. The downstream CpG island was chosen for promoter methylation analysis, since the shorter *DKK3 *transcript was shown to be more commonly distributed in normal human tissues [31].

### Bisulfite-modification and methylation-specific PCR

Approximately 1 μg of genomic DNA was bisulfite-modified using the EZ DNA Methylation Kit (Zymo Research, Orange, CA) according to the manufacturer's recommendations. The bisulfite-converted DNA was finally eluted in 20 μl of Tris buffer (10 mM; pH 7.6). Methylation-specific PCR was performed according to Herman *et al*. [32]. In short, 1 μl of modified DNA (~50 ng) was amplified using MSP primers (see Table 1) that specifically recognized either the unmethylated or methylated promoter sequences after bisulfite conversion [32]. Reaction volumes of 25 μl contained 1 × MSP-buffer [33], 400 nM of each primer, and 1.25 mM of each dNTP. One drop of mineral oil was added to each reaction tube. The PCR was initiated as "Hot Start" PCR at 95°C and held at 80°C before the addition of 1.25 units *Taq *DNA polymerase (Promega, Madison, WI). Cycle conditions were: 95°C for 5 min, 34 cycles of 95°C for 30 sec, 55°C (58°C) for 30 sec, 72°C for 40 sec and a final extension at 72°C for 5 min. Amplification products were visualized on 3% low range ultra agarose gel (Bio-Rad Laboratories, Hercules, CA) containing ethidium bromide and illuminated under ultraviolet light. Specificity of MSP primers in detecting the promoter methylation status were demonstrated by use of universal unmethylated and universal poly-methylated DNA as template (Epi Tect Control DNA Set; Qiagen, Hilden, Germany). Sensitivity of the utilized primers and cycling conditions was defined by use of a dilution series in MSP assays, constituted of methylated DNA diluted with unmethylated DNA (50%, 10%, 1%, 0.1%, 0.01% and 0% methylation).

**Table 1:** Oligonucleotide primers used in the study

	Sequence (5' → 3')	T_A_ [°C]	Primer [nM]	Product size (bp)
***Methylation-specific PCR***				

*WIF1 *unmethylated	Forward: GGGTGTTTTATTGGGTGTATTGT	55	400	154
	Reverse: AAAAAAACTAACACAAACAAAATACAAAC			

*WIF1 *methylated	Forward: CGTTTTATTGGGCGTATCGT	55	400	145
	Reverse: ACTAACGCGAACGAAATACGA			

*DKK3 *unmethylated	Forward: TTAGGGGTGGGTGGTGGGGT	58	320	126
	Reverse: CTACATCTCCACTCTACACCCA			

*DKK3 *methylated	Forward: GGGCGGGCGGCGGGGC	58	320	120
	Reverse: ACATCTCCGCTCTACGCCCG			

T_A_, annealing temperature.

### Statistical evaluations

Statistical analyses were completed using SPSS 14.0 (SPSS, Chicago, IL). Differences were considered significant when *P*-values were below 0.05. To study statistical associations between clinicopathological factors and methylation status contingency tables and two-sided Fisher's exact test were accomplished. In case of multiple statistical tests, the false discovery rate controlling procedure was applied. Survival curves were calculated using the Kaplan-Meier method, with significance evaluated by two-sided log-rank statistics. OS (n = 125) was measured from the day of surgery until breast cancer-related death (n = 21) and was censored for patients alive at last contact (n = 91), in case of death unrelated to the tumor (n = 5) or when the death cause was unknown (n = 8). DFS (n = 125) was measured from surgery until local or distant relapse (n = 30) and was censored for patients alive without evidence of relapse at the last follow-up (n = 95). A stepwise multivariate Cox regression model was adjusted, testing the independent prognostic relevance of clinical/investigational factors. The limit for reverse selection procedures was p = 0.1. Only patients for whom the status of all variables was known (n = 103) were included in the proportional hazard models. The proportionality assumption for all variables was assessed with log-negative-log survival distribution functions. The variables tumor size (pT), node status (pN) and histological grade (G) were dichotomised into less and more progressive groups (pT1-2 *vs*. pT3-4; pN0 *vs*. pN1-3; G1-2 *vs*. G3).

## Results

### *WIF1 *promoter methylation in primary breast carcinomas

**Figure 1:**
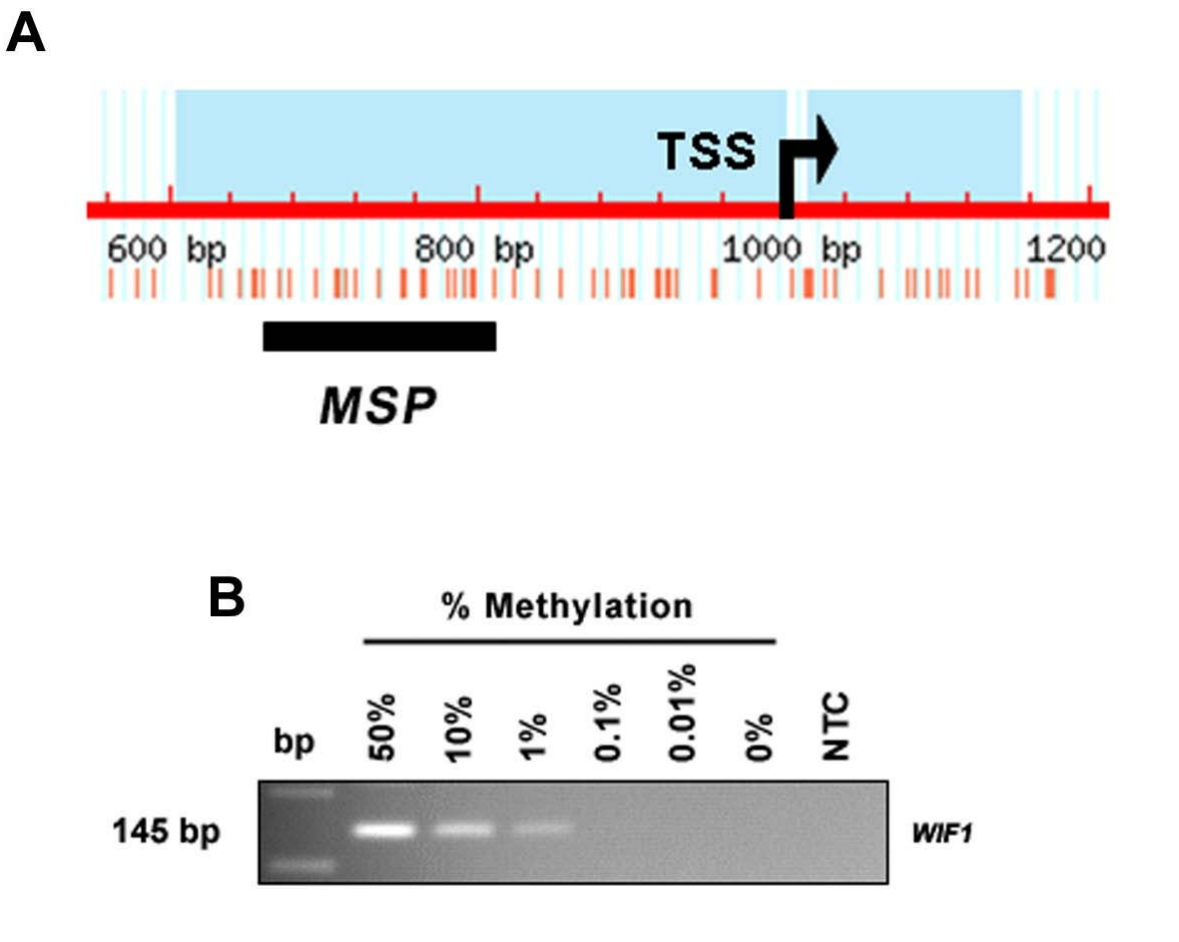
**Methylation analysis of the human *WIF1 *promoter**. (**A**) A 1.29 kb genomic sequence of the *WIF1 *promoter, analyzed by methprimer software [29], revealed the presence of a CpG island (blue) between relative position 604 and 1153. Position 1000 indicates the transcription start site (TSS, arrow). A region of high CpG (red vertical bars) densitiy was chosen for MSP analysis. The black bar indicates the MSP amplicon. (**B**) Sensitivity of the utilized MSP primers was determined by a dilution series of methylated DNA with unmethylated DNA (Epi Tect control DNA, Qiagen). At least 1% of methylated DNA (~0.1 ng) can be detected with the *WIF1 *MSP primers. bp, base pair marker; NTC, 'no template control'.

**Figure 2:**
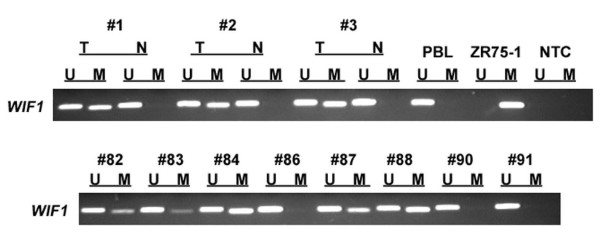
***WIF1 *methylation in primary breast cancer**. *WIF1 *methylation analyses of primary breast cancer specimens. MSP was performed on bisulfite-treated DNA from breast cancer (T) and matching normal primary breast tissues (N). MSP results from three representative matched pairs and eight additional breast carcinomas (#) are shown. DNA bands in lanes labeled with U indicate MSP products amplified with primers recognizing the unmethylated promoter sequence. DNA bands in lanes labeled with M represent amplified MSP products with methylation-specific primers. Peripheral blood lymphocytes (PBL) and breast cancer cell line ZR75-1 served as positive controls for the methylation-specific reaction, respectively. Water was used as template in the 'no template control' (NTC). Note that tumor tissue usually displayed a PCR product in the U-reaction as well, due to contaminating normal tissue (stromal cells, endothelial cells) present in the tumor specimens as has also been described by Suzuki *et al*. [34].

*WIF1 *promoter methylation in human breast cancer has been previously reported by Ai *et al*. [11], who demonstrated, by use of MSP, *WIF1 *methylation in 16 of 24 (67%) breast carcinoma samples. For three breast tumor specimens, these MSP results had also been confirmed by BGS. Unfortunately, in their study the *WIF1 *promoter regions investigated by MSP and BGS were not matching or overlapping, so we decided to analyze *WIF1 *promoter methylation in breast cancer by MSP in the particular promoter region that has been covered by BGS in the other study (Figure 1A). Initially, a dilution series of methylated DNA in an excess of unmethylated DNA (Epi Tect Control DNA, bisulfite-converted) was tested by MSP. This experiment determined the sensitivity of the utilized *WIF1 *MSP assay to be 1.0% in the detection of methylated DNA molecules (~0.1 ng) in a background of unmethylated DNA (~9.9 ng) (Figure 1B). Next, *WIF1 *promoter methylation was determined by MSP in 150 primary breast carcinoma specimens and also in 19 matching normal breast tissues. In all normal breast tissues, only unmethylated *WIF1 *promoter sequence could be detected, as indicated by exclusive amplification with primers recognizing the unmethylated DNA sequence (Figure 2). In contrast, 95 of 150 primary breast carcinomas (63.3%) revealed a methylated *WIF1 *promoter sequence, as indicated by amplification with primers specific to methylated DNA (Figure 2). The remaining 55 tumor specimens (36.7%) revealed solely unmethylated *WIF1 *promoter sequence. In general, tumor samples, despite methylation, also revealed unmethylated *WIF1 *promoter sequence, which is likely due to small contaminations with stromal and endothelial cells, as has also been previously described [34].

### *DKK3 *promoter methylation in primary breast carcinomas

**Figure 3:**
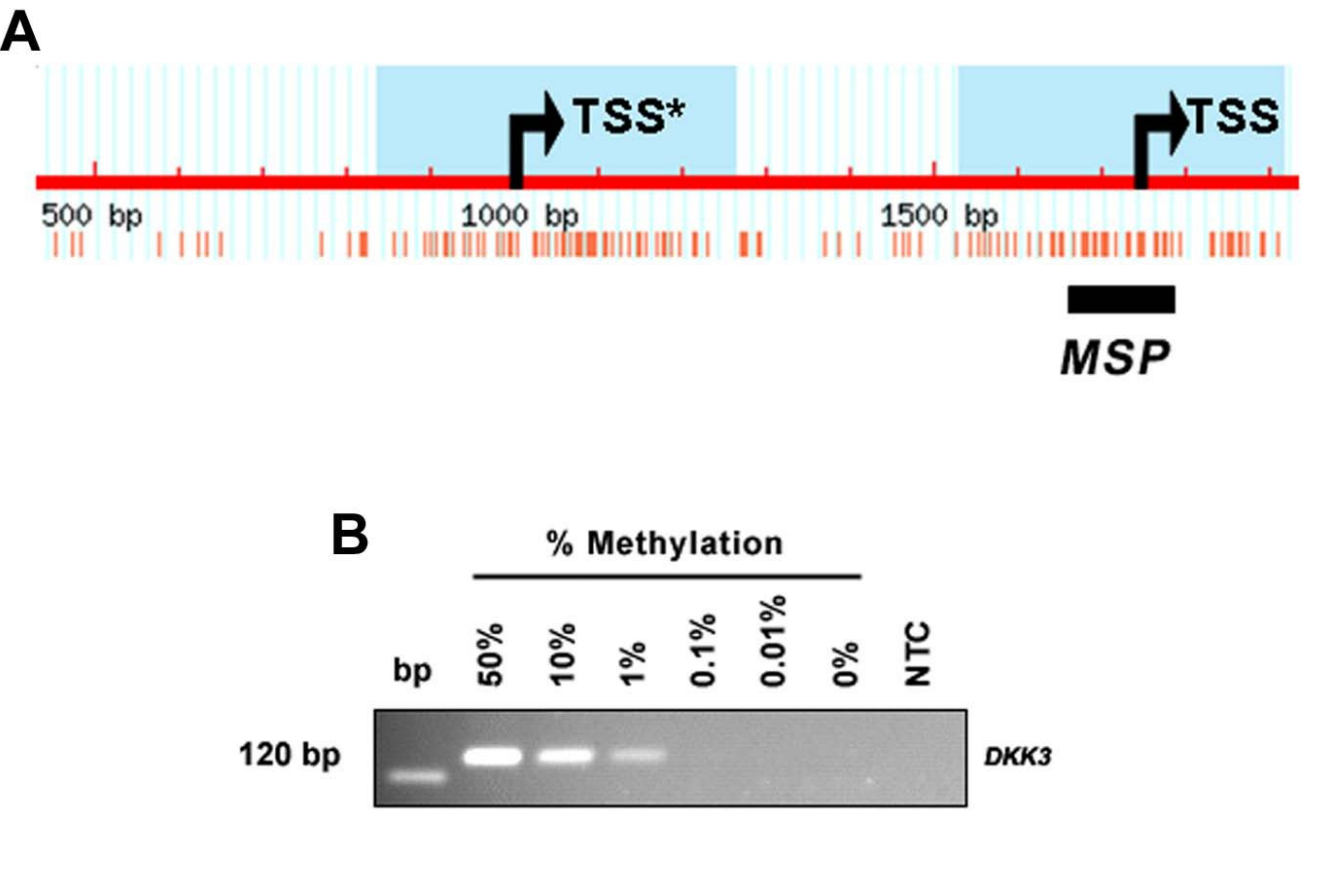
**Methylation analysis of the human *DKK3 *promoter**. (**A**) A 2.0 kb genomic sequence of the *DKK3 *promoter, analyzed by methprimer software [29], revealed the presence of two CpG islands (blue); one between relative position 834 and 1261 and another one between position 1529 and 1917. Two alternative tissue-specific *DKK3 *transcripts have been described [30]. Since transcription of the shorter transcript is widely distributed in normal tissues, we chose the region of the second transcription start site (TSS, arrow) for methylation analysis. Position 1000 indicates the alternative transcription start site of the longer transcript (TSS*, arrow). A region of high CpG (red vertical bars) densitiy within the second CpG island was chosen for MSP analysis. (**B**) Sensitivity of the utilized MSP primers was determined by a dilution series of methylated DNA with unmethylated DNA (Epi Tect control DNA, Qiagen). At least 1% of methylated DNA (~0.1 ng) can be detected with the *DKK3 *MSP primers. bp, base pair marker; NTC, 'no template control'.

We have recently reported of frequent *DKK3 *promoter methylation in human breast cancer [13]. In the respective report, we have demonstrated that *DKK3 *methylation was present in 61.3% of breast cancer patients (92 of 150), whereas in 19 matching normal breast tissues only one sample (5.3%) revealed faint methylation signals. Ideally, the samples analyzed for *WIF1 *methylation in the present report were physically identical with the samples previously analyzed for *DKK3 *methylation. The previously analyzed *DKK3 *promoter region is pictured in Figure 3A. To allow a direct comparison between methylation of these two genes, we first assayed a dilution series of methylated DNA with unmethylated DNA by MSP using *DKK3 *methylation-specific primers (see above). This experiment determined the sensitivity of the utilized *DKK3 *MSP assay to be 1.0% in the detection of methylated DNA molecules (~0.1 ng) in a background of unmethylated DNA (~9.9 ng) (Figure 3B), thus enabling a subsequent correlation analysis in breast cancer employing the methylation results from both genes.

### Association of *WIF1 *and *DKK3 *promoter methylation with clinicopathological parameters

For descriptive data analysis clinicopathological parameters were correlated with the *WIF1 *and *DKK3 *promoter methylation status. In a bivariate analysis, *WIF1 *methylation was not associated with patient age at diagnosis, tumor size, lymph node status, histological grade, histological type, and estrogen or progesterone receptor status (Table 2). *DKK3 *methylation was associated with advanced patient age at diagnosis (p = 0.007), but not associated with any other of the investigated parameters (Table 2).

**Table 2:** Demographic/clinicopathological parameters in relation to *WIF1 *and *DKK3 *promoter methylation

		*WIF1 *methylation				*DKK3 *methylation			
		
Variable	Categorization	n^1^	No (%)	Yes (%)	*P*^2^	n^1^	No (%)	Yes (%)	*P*^2^
***Clinicopathological factors***									

Age at diagnosis									
	<57 years	74	32 (43)	42 (57)	0.127	74	37 (50)	37 (50)	0.007
	≥ 57 years	76	23 (30)	53 (70)		76	21 (28)	55 (72)	

Tumor size^3^									
	pT1-pT2	129	48 (37)	81 (63)	1.000	129	52 (40)	77 (60)	0.440
	pT3-pT4	18	7 (39)	11 (61)		18	5 (28)	13 (72)	

Lymph node status^3^									
	pN0	72	29 (40)	43 (60)	0.606	72	32 (44)	40 (56)	0.170
	pN1-pN3	71	25 (35)	46 (65)		71	23 (32)	48 (67)	

Histological grade									
	G1-G2	88	28 (32)	60 (68)	0.170	88	31 (35)	57 (65)	0.313
	G3	62	27 (44)	35 (57)		62	27 (44)	35 (57)	

Histological type									
	IDC	122	43 (35)	79 (65)		122	45 (37)	77 (63)	
	lobular	19	7 (37)	12 (63)	0.296	19	7 (37)	12 (63)	0.236
	other	9	5 (56)	4 (44)		9	6 (67)	3 (33)	

*Immunohistochemistry*									

Estrogen receptor									
	negative (IRS^4 ^0–2)	47	21 (45)	26 (55)	0.142	47	20 (43)	27 (57)	0.467
	positive (IRS 3–12)	98	31 (32)	67 (68)		98	35 (36)	63 (64)	

Progesterone receptor									
	negative (IRS^4 ^0–2)	51	22 (43)	29 (57)	0.206	51	21 (41)	30 (59)	0.593
	positive (IRS 3–12)	94	30 (32)	64 (68)		94	34 (36)	60 (64)	

*WIF1 promoter*									

	unmethylated	-	-	-	-	55	29 (53)	26 (47)	0.009
	methylated	-	-	-		95	29 (31)	66 (69)	

^1^Only female patients with primary, unilateral invasive breast cancer were included. ^2^Fisher's exact test. ^3^According to UICC: TNM Classification of Malignant Tumours [25]. ^4^IRS, immunoreactivity score according to Remmele and Stegner [27]. Percentages may not sum to 100 due to rounding. IDC, invasive ductal carcinoma; n.a., not available.

### Correlation of *WIF1 *and *DKK3 *promoter methylation in primary breast carcinoma

**Figure 4:**
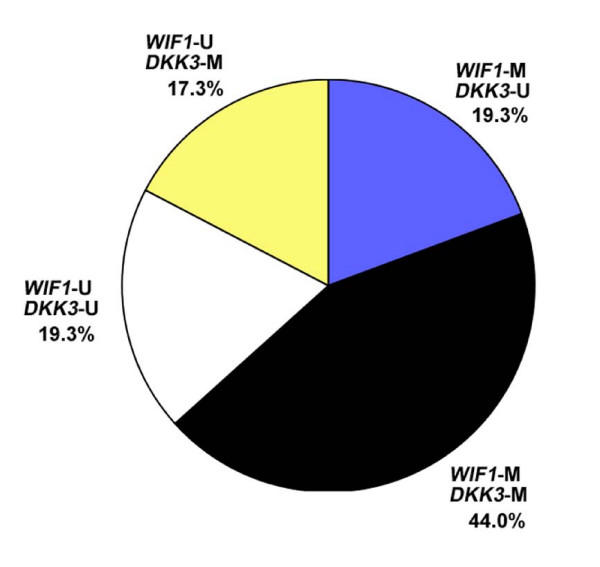
**Distribution of *WIF1 *and *DKK3 *promoter methylation in primary breast carcinomas**. Methylation status of either gene has been determined by MSP in the same tumors. Of n = 150 breast cancer patients, the larger fraction reveals an identical methylation status of both genes (63.3%). In the remaining smaller fraction (36.6%), methylation of only one of the two genes could be detected. In total, *WIF1 *methylation was significantly associated with *DKK3 *methylation (p = 0.009; Fisher's exact test).

In a bivariate analysis, methylation of the *WIF1 *promoter was significantly associated with methylation of the *DKK3 *promoter (p = 0.009) (Table 2). Both gene promoters were mutually unmethylated in tumors from 29 of 150 patients (19.3%) and mutually methylated in tumors from 66 of 150 patients (44.0%) (Figure 4). For 55 of 150 patients (36.7%) the methylation status of the *WIF1 *and *DKK3 *promoter differed: *WIF1 *methylation together with *DKK3 *non-methylation was detected in 29/250 patients (19.3%), whereas *WIF1 *non-methylation together with *DKK3 *methylation was detected in 26 of 150 patients (17.3%).

### Association of *WIF1 *promoter methylation with patient survival

**Figure 5:**
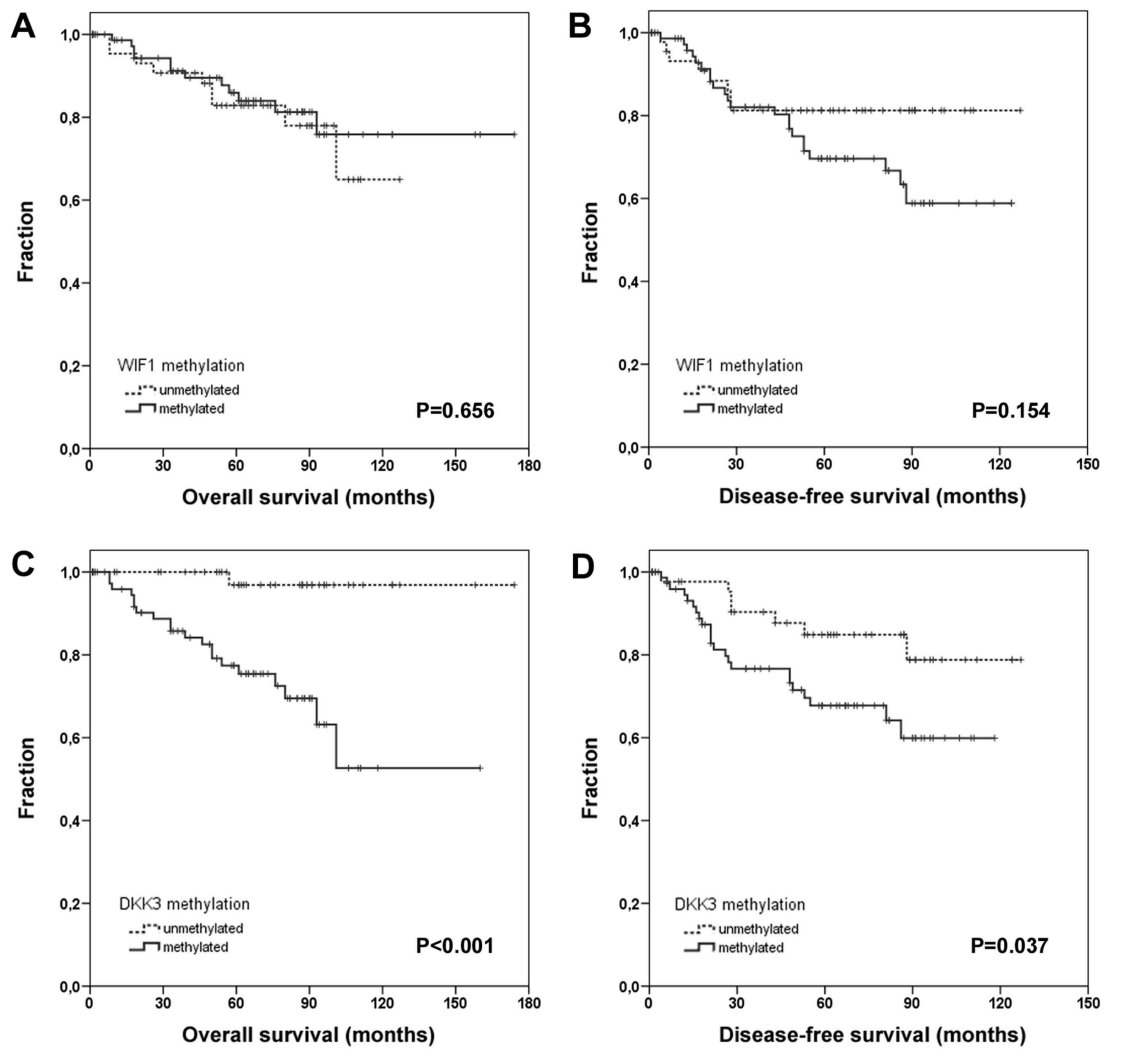
**Univariate Kaplan-Meier survival analysis of breast cancer patients in relation to *WIF1 *and *DKK3 *promoter methylation**. (**A**) Overall survival and (**B**) disease-free survival are not associated with *WIF1 *promoter methylation in human breast cancer. Solid lines indicate methylated *WIF1 *promoter; dotted lines indicate unmethylated *WIF1 *promoter in the tumor. (**C**) In contrast, methylation of the *DKK3 *promoter in tumor tissue (solid line) is significantly associated with adverse patient overall survival, whereas patients with an unmethylated *DKK3 *promoter in the tumor tissue have a very favorable clinical outcome (dotted line) (p < 0.001). (**D**) In addition, *DKK3*-methylated tumors reveal a significant shorter time to recurrence (solid line), as compared to tumors harboring an unmethylated *DKK3 *promoter (dotted line) (p = 0.037). Vertical tick marks represent censored patients.

Patient OS and DFS were compared between methylated *versus *unmethylated *WIF1 *promoter sequence by univariate Kaplan-Meier analysis using log-rank statistics. In this analysis, *WIF1 *methylation was not significantly associated with patient OS (p = 0.656) or patient DFS (p = 0.154) (Table 3), as also demonstrated by Kaplan-Meier survival curves (Figure 5). As expected, a positive lymph node status (pN1-3) and higher histological grade (G3) were found to be associated with decreased OS (p = 0.002; p = 0.001) and DFS (p < 0.001; p = 0.012).

**Table 3:** Univariate survival analysis of clinicopathological and molecular factors (log-rank test)

Variable	Categorization	Overall survival			Disease-free survival		
		
		n^1^	events	*P*^2^	n^1^	events	*P*^2^
***Clinicopathological factors***							

Age at diagnosis							
	<57 years	64	7	0.094	64	16	0.711
	≥ 57 years	61	14		61	14	

Tumor size^3^							
	pT1-pT2	107	17	0.372	107	25	0.427
	pT3-pT4	16	4		16	5	

Lymph node status^3^							
	pN0	54	3	0.002	54	5	<0.001
	pN1-pN3	65	18		65	24	

Histological grade							
	G1-G2	72	5	0.001	72	11	0.012
	G3	53	16		53	19	
Histological type							
	IDC	101	19	0.267	101	22	0.277
	other	24	2		24	8	

*Immunohistochemistry*							

Estrogen receptor							
	negative (IRS^4 ^0–2)	40	9	0.155	40	9	0.962
	positive (IRS 3–12)	80	12		80	21	

Progesterone receptor							
	negative (IRS^4 ^0–2)	39	9	0.154	39	13	0.087
	positive (IRS 3–12)	81	12		81	17	

*WIF1 promoter*							

	unmethylated	47	9	0.656	47	8	0.154
	methylated	78	12		78	22	

*DKK3 promoter*							

	unmethylated	46	1	<0.001	46	7	0.037
	methylated	79	20		79	23	

^1^Only female patients with primary, unilateral invasive breast cancer were included. ^2^Log-rank test. ^3^According to UICC: TNM Classification of Malignant Tumours [25]. ^4^IRS, immunoreactivity score according to Remmele and Stegner [27]. IDC, invasive ductal carcinoma.

### Association of *DKK3 *promoter methylation with patient survival

Patient OS and DFS were compared between methylated *versus *unmethylated *DKK3 *promoter sequence. In contrast to *WIF1 *methylation, *DKK3 *methylation was significantly associated with poor OS (5-year survival: 75% for cases with methylated alleles *vs*. 97% for cases with unmethylated alleles; 10-year survival: 54% *vs*. 97%; p = 0.0005; Table 3) and shorter DFS (5-year survival: 67% for cases with methylated alleles *vs*. 84% for cases with unmethylated alleles; 10-year-survival: 58% *vs*. 78%; p = 0.037; Table 3), as also illustrated by Kaplan-Meier survival curves (Figure 5). Based on a mean OS of 141 months (95% CI: 127–154 months) patients without *DKK3 *methylation in the tumor tissue revealed much longer mean OS (170 months, 95% CI: 163–177 months) than patients with *DKK3 *methylation in the tumor tissue (113 months, 95% CI: 95–131 months). Based on a mean DFS of 99 months (95% CI: 90–107 months) patients without *DKK3 *methylation in the tumor revealed longer mean DFS (110 months, 95% CI: 99–122 months) than patients with *DKK3 *methylation in the tumor (86 months, 95% CI: 75–97 months). Multivariate Cox regression models were calculated and adjusted to assess factor-related hazard risks and to test for independency of *DKK3 *methylation as a prognostic factor in patient OS and DFS. The strength of the association between *DKK3 *methylation and unfavourable patient outcome is presented in Table 4 and Table 5. Multivariate, *DKK3 *methylation in breast carcinoma represented an independent and strong risk factor for OS (HR: 14.4; 95% CI: 1.9 – 111.6; p = 0.011; Table 4). In DFS the prognostic potency of *DKK3 *methylation was weaker than in OS (HR: 2.5; 95% CI: 1.0 – 6.0; p = 0.047; Table 5).

**Table 4:** Multivariate Cox regression analysis of *DKK3 *promoter methylation with regard to overall survival

			Multivariate analysis Overall survival (global model)			Multivariate analysis Overall survival (reverse selection procedure^2^)		
**Variable**			**HR**	**95% CI^1^**	***P***	**HR**	**95% CI^1^**	***P***

Age at diagnosis	<57 years	0	1.0			1.0		
	≥ 57 years	1	1.78	0.63 – 4.99	0.276	2.27	0.85 – 6.07	0.104

Tumor size	pT1-2	0	1.0					
	pT3-4	1	0.83	0.25 – 2.78	0.766			

Lymph nodes	pN0	0	1.0			1.0		
	pN1-3	1	5.47	1.46 – 20.53	0.012	4.50	1.26 – 15.87	0.021

Histological grade	G1	0	1.0			1.0		
	G2-G3	1	4.36	1.54 – 12.40	0.006	4.50	1.57 – 12.87	0.005

Histological type	ductal	0	1.0					
	other	1	0.46	0.09 – 2.22	0.330			

Estrogen receptor	negative	0	1.0			1.0		
	positive	1	0.51	0.18 – 1.47	0.214	0.43	0.17 – 1.09	0.426

Progesterone receptor	negative	0	1.0					
	positive	1	0.57	0.21 – 1.54	0.270			

*DKK3 *promoter	unmethylated	0	1.0			1.0		
	methylated	1	14.41	1.86 – 111.56	0.011	13.68	1.77–105.52	0.012

^1^Confidence interval (CI) on the estimated hazard ratio (HR). ^2^Only terms that remained in the model after reverse selection are listed. All variables were stratified binary according to Table 3.

**Table 5:** Multivariate Cox regression analysis of *DKK3 *promoter methylation with regard to disease-free survival

			Multivariate analysis Disease-free survival (global model)			Multivariate analysis Disease-free survival (reverse selection procedure^2^)		
**Variable**			**HR**	**95% CI^1^**	***P***	**HR**	**95% CI^1^**	***P***

Age at diagnosis	<57 years	0	1.0					
	≥ 57 years	1	0.57	0.24 – 1.33	0.191			

Tumor size	pT1-2	0	1.0					
	pT3-4	1	0.61	0.22 – 1.72	0.353			

Lymph nodes	pN0	0	1.0			1.0		
	pN1-3	1	4.24	1.50 – 11.96	0.006	4.01	1.49 – 10.81	0.006

Histological grade	G1	0	1.0			1.0		
	G2-G3	1	2.01	0.91 – 4.43	0.086	1.93	0.88 – 4.24	0.102

Histological type	IDC	0	1.0					
	other	1	1.04	0.42–2.62	0.929			

Estrogen receptor	negative	0	1.0					
	positive	1	2.17	0.78 – 6.03	0.137			

Progesterone receptor	negative	0	1.0			1.0		
	positive	1	0.36	0.15 – 0.88	0.025	0.53	0.25 – 1.11	0.091

*DKK3 *promoter	unmethylated	0	1.0			1.0		
	methylated	1	2.46	1.01 – 5.97	0.047	2.08	0.88 – 4.88	0.094

^1^Confidence interval (CI) on the estimated hazard ratio (HR). ^2^Only terms that remained in the model after reverse selection are listed. All variables were stratified binary according to Table 3.

## Discussion

It was previously reported that expression of the Wnt antagonist genes *WIF1 *and *DKK3 *is downregulated in several tumor entities as a consequence of epigenetic DNA modification [11,13,21,31,35,36]. WIF1 is a conserved Wnt-binding protein that prevents Wnt ligands from interacting with membranous frizzled receptors, thus may inhibit activation of the Wnt/β-catenin signaling cascade [37]. In breast, lung, prostate and bladder cancer, WIF1 expression was found to be frequently downregulated [38], suggesting it might represent a tumor suppressor gene. In breast cancer, this downregulation could be attributed to hypermethylation of the *WIF1 *promoter [11], as demonstrated both in breast cell lines and in primary breast carcinomas. In our study, methylation of the *WIF1 *promoter was detected in 63% of invasive tumors from breast cancer patients, thus being in good agreement with previous results from Ai *et al*. [11], who reported a frequency of 67% for *WIF1 *methylation in mammary tumors. Differences may arise through different sample sizes (n = 150 and n = 24) as well as different promoter locations assessed in either study. DKK3 is a further secreted inhibitor of Wnt signaling, but in contrast to WIF1 does not sequester Wnt ligands. The actual mechanism by which DKK3 acts inhibitory on Wnt pathway activation has not been identified yet, but suppression of DKK3 increased β-catenin/T-cell factor (TCF)-dependent gene activity in mammary cells [39], cancerous lung cells [40] and glioma [41]. Likewise to *WIF1*, the *DKK3 *gene was also reported as a frequent target of epigenetic inactivation in numerous tumor entities, *e.g*. in lung cancer, prostate cancer and leukemia [21,31,42], suggesting that DKK3 may exert tumor suppressive functions. In a recent report, we have demonstrated that *DKK3 *is frequently inactivated in invasive breast carcinomas by promoter methylation leading to loss of DKK3 expression [13]. This epimutation affected 92 of 150 investigated breast cancer patients (61%). Since these samples were identical to the samples for which we now have determined *WIF1 *methylation, we were able to perform a combined analysis of both genes' methylation in breast cancer.

In a bivariate analysis, *WIF1 *methylation status in breast carcinomas was significantly associated with the *DKK3 *methylation status. Despite, within the cohort of carcinomas being affected by methylation of either the *DKK3 *or *WIF1 *gene, a large fraction (45%) showed methylation in one gene only. This demonstrates that in spite of a statistical association between methylation of the two genes, there is still a large fraction of breast cancer patients with different *DKK3/WIF1 *methylation pattern. Therefore, it is unlikely that methylation of both Wnt antagonist genes is a mandatory mutual carcinogenic event. In a further correlation analysis, neither of the two genes was associated with relevant clinicopathological features, except for *DKK3 *methylation being associated with advanced patient age. Age-dependent promoter methylation has been reported previously [43,44] and may randomly overlay the effects of gene-specific promoter methylation that can lead to the development of distinct cancer subtypes. The absence of an association between *WIF1 *or *DKK3 *methylation with important clinicopathological factors like tumor size, histological grade and lymph node invasion strongly suggests that methylation of either gene is an early carcinogenic event in breast cancer development, rather than contributing to tumor progression.

Most important, major differences between *WIF1 *and *DKK3 *methylation arise in their association with breast cancer patient survival. *WIF1 *methylation showed no significance in clinical patient outcome in contrast to *DKK3 *methylation, which was tightly associated with adverse patient OS and weaker with short DFS in our study. Patients harboring *DKK3 *methylation in the tumor had a poor prognosis (54% chance of 10-years OS) in contrast to patients retaining an unmethylated *DKK3 *promoter, who had a favorable prognosis (97% chance of 10-years OS). This finding was supported by a multivariate Cox regression analysis in which *DKK3*-methylated patients revealed a high risk of tumor-related death (HR: 14.4). Hence, this parameter outperformed classical prognostic factors in our patient cohort, *i.e*. high histological grade (HR: 4.4) or a positive lymph node status (HR: 5.5). Unproportional HRs of high impact were rarely achieved even in studies with very large sample size numbers, neither by strong conventional factors like node status (HR: 2.4) [45] and grade (HR: 5.7) [46] nor by investigational factors like the tissue urokinase-type plasminogen activator/inhibitor (uPA/PAI), which in case of high level exposes patients to a five times greater risk of dying from breast cancer [47]. In conclusion, our results demonstrate that determination of the *DKK3 *methylation status may provide valuable information to aid prognostication in the clinical management of breast cancer patients. Notably, methylation of the *DKK3 *promoter was recently shown to be prognosis relevant also in other tumor entities, such as in acute lymphoblastic leukemia, kidney cancer, lung cancer, and gastric cancer [21-24], pointing to a potential clinical use of this marker in several cancer diseases.

Our findings raise expectations towards translation of such methylation markers into clinical practice. As an example, *DKK3 *may be a prime candidate gene to be incorporated into diagnostic multimarker panels, for its aberrant methylation is specific to malignant cells in breast cancer [13]. Preliminary results from our laboratory revealed that *DKK3 *methylation can be detected with high clinical sensitivity and specificity in blood serum of breast cancer patients independent of tumor size and node status (unpublished data). The presence of detectable tumor DNA in serum is generally associated with poor prognosis [48,49], and taken together with its marker performance in solid breast tumor tissue, *DKK3 *methylation fulfils essential prerequisites as a biomarker in a blood-borne assay, of which we will report in a future study.

In summary, we here demonstrate that although *WIF1 *and *DKK3 *promoter methylation are similar frequent alterations in human breast cancer, only *DKK3 *methylation appears to be a survival risk factor for breast cancer patients and thus might be useful as prognostic marker in clinical oncology helping to improve patient outcome.

## Conclusion

This study shows that the Wnt antagonist gene *WIF1 *is frequently inactivated by promoter hypermethylation in human breast cancer. Although *WIF1 *is similarly frequent hypermethylated like the Wnt antagonist gene *DKK3*, and neither gene methylation is associated with relevant clinicopathological factors, *DKK3 *methylation is an independent prognostic factor in breast cancer patient survival, whereas *WIF1 *methylation is not. These differences may reflect subtle distinctions in the biological roles of the two related molecules in inhibiting Wnt/β-catenin signaling.

## Abbreviations

BGS: bisulfite genomic sequencing; BMBF: Bundesministerium für Bildung und Forschung; CI: confidence interval; DFS: disease-free survival; DKK3: Dickkopf-3; ER: estrogen receptor; HR: hazard ratio; MSP: methylation-specific polymerase chain reaction; NTC: no template control; OS: overall survival; PCP: planar cell polarity pathway; PCR: polymerase chain reaction; PR: progesterone receptor; TCF: T-cell factor; UICC: International Union Against Cancer; UMD: universal methylated DNA; UUD: universal unmethylated DNA; WHO: World Health Organization; WIF1: Wnt-inhibitory factor 1.

## Competing interests

ED has declared that he has submitted a patent application on the use of *DKK3 *promoter methylation. The other authors declare that they have no competing interests.

## Authors' contributions

JV carried out the methylation experiments, contributed to statistical evaluations, participated in the conception and design of the study, and wrote the manuscript. PJW participated in statistical analyses and data interpretation, and critically revised the manuscript. TF and PJS supported with expertise in statistical analyses, and critically revised the manuscript. AH provided clinical samples and clinicopathological data, and critically revised the manuscript. RK participated in the design and coordination of the study, and critically revised the manuscript. ED planned and coordinated the study, and critically revised the manuscript. All authors have given final approval of the version to be published.

## Pre-publication history

The pre-publication history for this paper can be accessed here:

http://www.biomedcentral.com/1471-2407/9/217/prepub
